# Image quality and radiation dose of dual source high pitch computed tomography in pediatric congenital heart disease

**DOI:** 10.1038/s41598-022-13404-w

**Published:** 2022-06-15

**Authors:** Dmitrij Kravchenko, Christopher Hart, Stephan Garbe, Julian A. Luetkens, Alexander Isaak, Narine Mesropyan, Mathieu Vergnat, Judith Leyens, Ulrike Attenberger, Daniel Kuetting

**Affiliations:** 1 Department of Diagnostic and Interventional Radiology, University Hospital Bonn, Venusberg-Campus 1, 53127 Bonn, Germany; 2 Quantitative Imaging Lab Bonn (QILaB), University Hospital Bonn, Bonn, Germany; 3 Department for Pediatric Cardiology, Children’s Hospital, University of Bonn, Bonn, Germany; 4 Department of Pediatric Cardiothoracic Surgery, Children’s Hospital, University of Bonn, Bonn, Germany; 5 Department of Neonatology and Pediatric Intensive Care, Children’s Hospital, University of Bonn, Bonn, Germany

**Keywords:** Cardiology, Medical research, Paediatric research

## Abstract

To explore the image quality and radiation dose of dual source high-pitch cardiac computed tomography with tailored contrast injection protocols for pediatric congenital heart disease patients (CHD). In total, 27 infants with CHD (median age 109 days [IQR 6–199]) were retrospectively analyzed regarding dose length product (DLP) and effective dose (ED) after undergoing cardiothoracic CT imaging. Scan parameters were adjusted on a dual source/detector CT (DSCT) to minimize radiation dose while maintaining adequate quality. Image acquisition was performed at 70% of the R–R interval. Dose reducing measures included prospective electrocardiogram gating, utilizing slow injection velocities and foregoing bolus tracking during contrast injection. Image quality was assessed for artefacts, vessel definition, and noise on a 5-point scale (1 non-diagnostic, 5 excellent). Series were scored on a 0-to-3-point scale regarding answered clinical questions (0 non-diagnostic, 3 all clinical questions could be answered). The median DLP was 5.2 mGy*cm (IQR 3.5–7.8) leading to a median ED of 0.20 mSv (IQR 0.14–0.30). On average the acquired images scored 13.3 ± 2.1 (SD) out of a maximum 15 points with an intraclass correlation coefficient (ICC) of 0.94. All acquired series were able to fully answer all clinical questions scoring maximum points (ICC 1.0). Dual source high pitch CT protocols combined with custom contrast agent injection protocols in pediatric patients with CHD delivered sufficiently high diagnostic imaging quality combined with low submilisievert radiation doses. Prospective high pitch imaging is a reliable method for depiction of cardiac anatomy even in very young pediatric CHD patients with elevated heart rates.

## Introduction

Congenital heart disease (CHD) is one of the most common causes of congenital abnormalities in the world with an estimated prevalence of 0.8–1.5%^1^. Detailed and extensive visualization of the anatomy of CHD is vital for postnatal care and therapeutic planning. While transthoracic echocardiography is the first choice due to its wide availability and lack of ionizing radiation, it can be limited in the visualization of thoracic vasculature and is dependent on user experience.

Current multi-detector computed tomography (CT) technology is increasingly being employed to visualize postnatal CHD patients^2^. CT imaging provides a non-invasive visualization technique with an adequate resolution, offers three-dimensional reconstruction possibilities, and is widely available. Additionally, it provides a more detailed overview of anatomic structures than echocardiography allowing for a greater spectrum of incidental findings.

As such, the need to develop cardiac computed tomography (CCT) protocols with low effective doses has become increasingly important. Dual source/detector CT scanners (DSCT) offer an important advantage over single detector scanners due to their drastically improved temporal resolution. In theory this enables single phase prospective imaging consecutively allowing for dose reduction while maintaining image quality.

Currently there are two mainstream ways to acquire pediatric CCTs: retrospective and prospective gating. Both methods utilize electrocardiograms (ECG) to synchronize the scan to the beating heart. Retrospective gating synchronizes images at the reconstruction stage after acquiring multiple cycles of the heartbeat. Prospective gating synchronizes during acquisition. While prospective gating emits radiation only during the to be acquired phase, retrospective gating irradiates the patient during multiple cycles leading to doses around 12.3 mSv^3^. The obvious advantage of prospective gating is the lower radiation dose, with effective doses in the range of 1.4–3.5 mSv^3,4^ and recently even sub-millisievert doses have been reported^5^. Developments in DSCT technology, including ultra-high pitch and wider detectors enable refined single cycle prospective gating. As a result, overscan and thus effective doses can be reduced.

Unfortunately, the drawbacks of CT imaging are self-evident. Especially in a young population, the use of CT imaging has been very reserved due to ionizing radiation and the associated increased risk of induced malignancies. The physiology of newborns itself presents with difficulties. For one, breath-holding is not feasible thus leading to additional motion artefacts. Secondly, the physiologic rapid heart rate also leads to motion artefacts as well as synchronization problems which can produce stepping artefacts depending on how the scan is performed.

The purpose of this study was to evaluate image quality and effective doses of prospective ECG gated, high pitch 196 slice DSCT in visualizing cardiac and extracardiac anomalies of CHD patients.

## Materials and methods

This study complies with the Declaration of Helsinki and all methods were performed in accordance with relevant guidelines and regulations. Study design and information processing was approved by the local institutional review board of the University Hospital Bonn (Ethics Committee of the Medical Faculty of the University of Bonn, Application number 382/21). Due to the retrospective nature of this study, patient informed consent was waived by the institutional review board (Ethics Committee of the Medical Faculty of the University of Bonn, Application number 382/21). Pediatric patients who underwent cardiothoracic CT imaging between February 2020 and August 2021 for any reason were screened. In total, 27 patients diagnosed with a CHD were included for analysis. Inclusion criteria included age under 365 days for better comparability to other studies who also used the specific conversion coefficient k for the 32 cm chest phantom for children aged one years or younger. Further inclusion criteria included diagnosis of CHD, defined as any congenital heart defect definitely or potentially requiring surgical intervention necessitating CT imaging for potential preoperative planning, and having received a DSCT under standardized scanning conditions. Exclusion criteria included DSCT imaging for other reasons than CHD or single source CT imaging. Patient characteristics are summarized in Table 1.

**Table 1:** A brief overview of the patient characteristics.

Characteristic	Values
Sex	
Females, n (%)	6 (22%)
Males, n (%)	21 (78%)
Age, days, median (IQR)	106 (6–199)
Height, cm	58.5 (7.6)
Weight, kg	5.0 (1.7)
BMI, kg/m^2^	14.2 (1.7)
Surface area (Mostellar), m^2^	0.28 (0.06)
Congenital heart defect	n (%)
HLHS: Hypoplastic left heart syndrome	6 (22%)
Pulmonary atresia	5 (19%)
TOF: Tetralogy of Fallot	5 (19%)
TAPVR: Total anomalous pulmonary venous return	4 (15%)
DORV: Double outlet right ventricle	4 (15%)
Single coronary ostium	1 (4%)
TGA: Transposition of the great arteries	1 (4%)
ALVT: Aorto-left ventricular tunnel (ALVT)	1 (4%)

All values are expressed as mean with standard deviation in brackets unless otherwise noted.

*IQR* Interquartile range, *BMI* Body mass index.

### DSCT parameters

All imaging was performed using a 2 × 192-slice third generation dual source CT (Somatom Force, Siemens Healthineers, Forchheim, Germany). Prospective “FLASH” ECG gating along with tube-current modulation in the angular and longitudinal direction (CareDose 4D, Siemens proprietary technology) was employed. All scans were acquired with 70 kVp. The pitch was set to 3.2 with a collimation of 192 × 0.6 mm in the craniocaudal direction. All scans were performed in free breathing.

### Image acquisition

Iodinated contrast medium, Accupaque 300 (Iohexol 647 mg/ml, GE, Boston, Massachusetts, USA) was injected either via central or peripheral vein cannula. Contrast medium protocols were individualized for each patient depending on the indication for the CT scan and the suspected underlying anatomy. Patients typically received 2 ml of contrast medium per kilogram of bodyweight, diluted with saline (1:1) injected at a rate between 0.5 and 1 ml per second, unless solely arterial contrast was required. CT scans were initiated depending on injection site, injection velocity, duration of injection, and expected circulation time. Scan range typically extended from above the subclavian veins to the hepatic vein confluence, to depict the entire thoracic vasculature. The high pitch mode was used alongside prospective ECG gating for image acquisition. Imaging was commenced at 70% of the R–R interval. Images were acquired using 0.6 mm slice thickness at 0.4 mm intervals and reconstructed utilizing a vascular kernel. Detailed scan parameters are outlined in Table 2. Beta blockers were not employed for heart rate control. Image acquisition was performed under sedation.

**Table 2:** Dual source computed tomography scan parameters for pediatric congenital heart disease patients.

Parameter	Value
kVp	70 kV
Reference mAs	Dose modulation: median 147 mAs (IQR: 105–210)
Pitch	3.2
Gantry rotation time	0.28 s
Table speed	737 mm/s
Detector collimation	192 × 0.6 mm
Slice collimation	0.6 mm
Convolution kernel	Bv40d\3
Slice thickness	0.6 mm
Interval	0.4 mm
Software	IMPAX EE R20 XIX SU1, Dedalus

*kVp* Kilovolt peak, *mAs* Milliampere seconds, *IQR* Interquartile range.

### Image quality assessment

Two experienced radiologists (DKu, 7 years of pediatric cardiac CT experience and DKr, 3 years of pediatric cardiac CT experience) analyzed the datasets. Images were reconstructed in 1 and 0.6 mm slice thickness utilizing a medium sharp vascular kernel (Siemens Healthineers Bv40d convolution kernel). Image analysis was performed using a standardized workstation running DeepUnity software (Dedalus Healthcare, DeepUnity R20 Bonn, Germany). The image quality score was assessed on a 5-point scale for three categories adapted from a previous study by Saad et al.^6^. Three categories were scored for each dataset: artefacts, noise, and vessel delineation. A maximum of 5 points per category were allocated as follows for a maximum score of 15 and a minimum score of 3:

1. Non-diagnostic: severe artefacts rendering images not diagnostic, too much noise to adequately separate anatomical structures, coronary arteries not visible.
2. Poor quality: severe artefacts, excessive noise, questionable coronary artery delineation.
3. Adequate quality: moderate artefacts, moderate noise, proximal coronary arteries are delineated.
4. Good quality: minor artefacts, low noise, coronary arteries clearly delineated.
5. Excellent quality: no artefacts, no noise, clear visualization of distal coronary artery segments.

**Figure 1:**
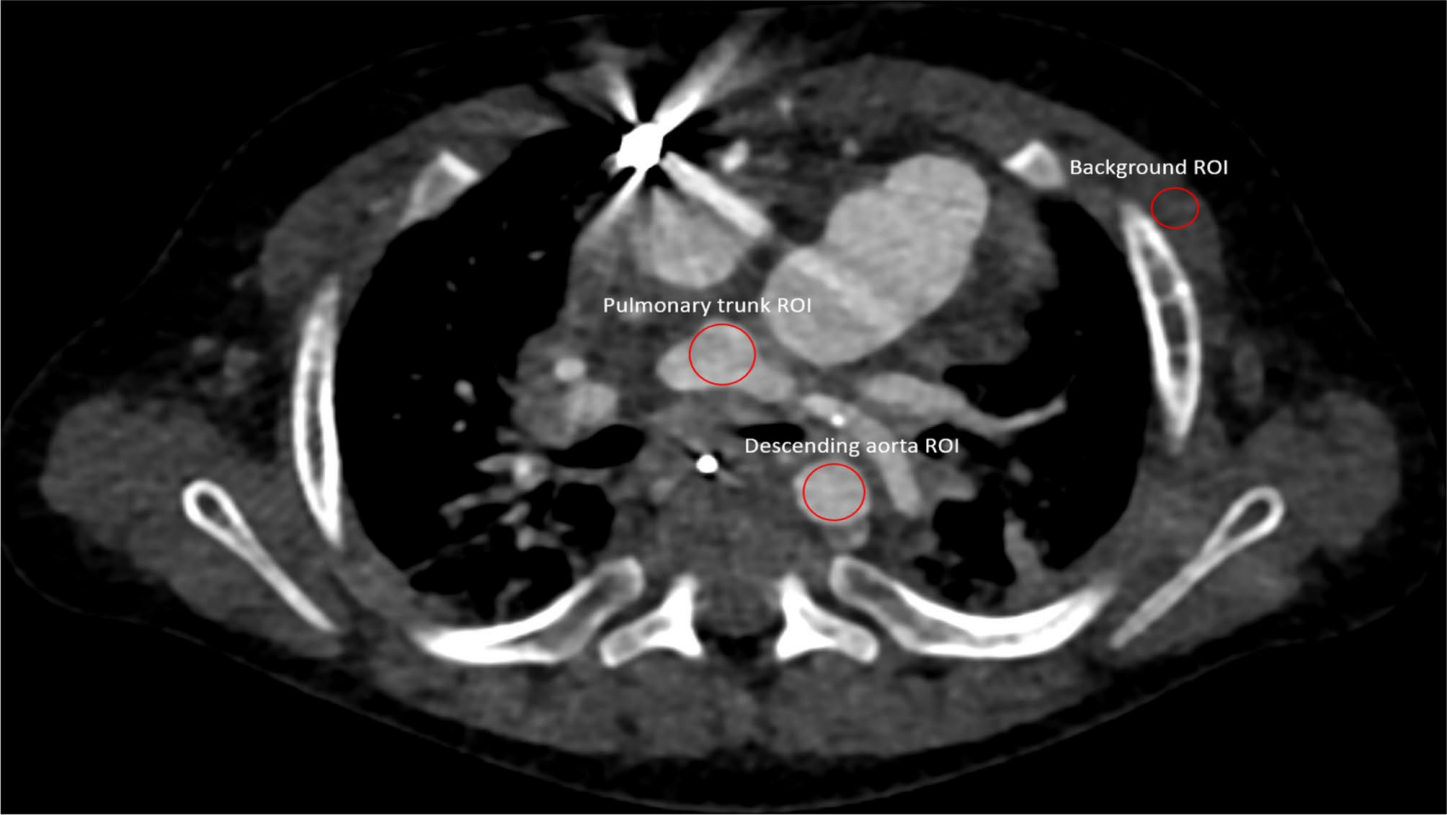
Region of interest (ROI) placement demonstrated in a 7-month-old male patient with hypoplastic left heart syndrome. Due to the varying anatomy of the included patients, ROI size was scaled individually for each patient to include as much reasonably possible without measuring adjacent tissues.

Additionally, SNR and contrast to noise ratio (CNR) were calculated as previously described (depicted in Eqs. 1 and 2)^7^. Regions of interest (ROI) were placed in the pulmonal trunk at the site of bifurcation, the descending aorta at the same height as the pulmonal trunk and muscle tissue at the same height as the pulmonal trunk. Pectoral muscles were used the background standard for the calculations. Due to the varying age and primary pathologies of the included patients, ROI size was scaled for each patient individually to include as much tissue as was available at the predetermined height as demonstrated in Fig. 1. All series were additionally rated to assess whether they sufficiently answered all clinical questions based on a numeric scale with 0 being non-diagnostic series, 1 some questions were answered, 2 most questions were answered, and 3 all questions were sufficiently answered.

1 $$ SNR = \frac{{HU_{ROI} }}{{\sigma_{ROI} }} $$

2 $$ CNR = \frac{{HU_\text{Object of Interest} - HU_{Background} }}{{\sigma_{Background} }}$$

### Dose estimates

Volume CT dose index (CTDIvol) was calculated by the CT console using a 32 cm Phantom. Dose length product (DLP) was also calculated by the CT console by multiplying the CTDIvol value with the distance in cm scanned. The DLP value was multiplied by the specific conversion coefficient (k) for the 32 cm chest phantom (k = 0.039 mSv/(mGy*cm) for children under one year old^8–10^. Equation 3 was used for the estimation of effective dose^11^.

3 $$ ED \left( {{\text{mSv}}} \right) = DLP \left( {{\text{mGy}}*{\text{cm}}} \right)*k\left( {{\text{mSv}}*{\text{mGy}}^{ - 1} *{\text{cm}}^{ - 1} } \right) $$

### Statistics

Statistical analysis was performed by Jamovi Version 1.6 (The Jamovi Project, Sydney, Australia). Results were expressed as means and standard deviations for quantitative variables and as frequencies or percentages for categorical variables. The Shapiro Wilks test was performed to assess normality. If normality could not be assumed, median and IQR were provided while significance was checked via the Mann Whitney U test. Interobserver agreement on grades of image quality was assessed by the intra-class correlation coefficient (ICC; < 0.5 poor, 0.5–0.75 moderate, 0.75–0.9 good, > 0.9 excellent reliability). Correlations between the score quantifying image quality, and cardiac parameters, reference mAs were calculated using the Pearson correlation coefficient. *P* values < 0.05 were considered statistically significant.

## Results

**Figure 2:**
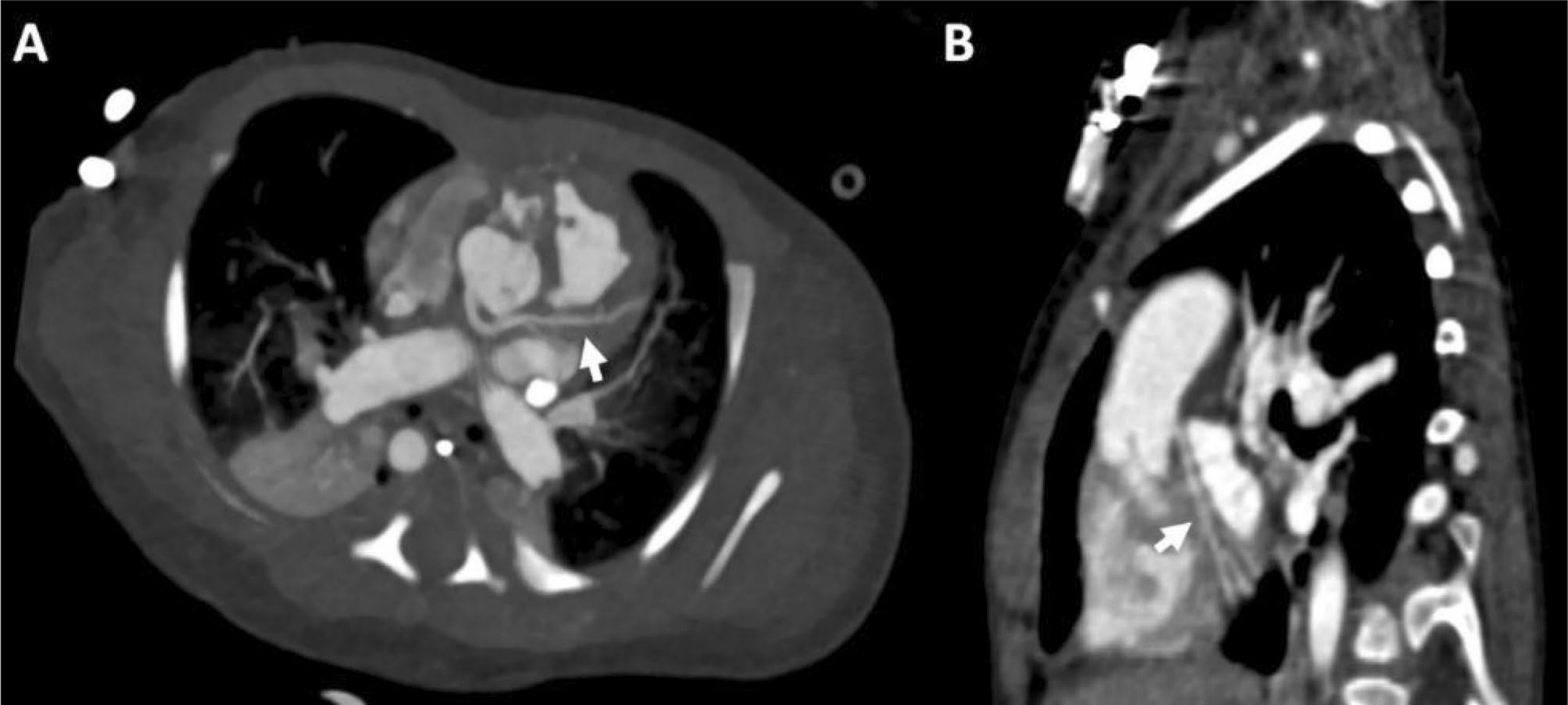
Double angulated 3D reconstructions of the left anterior descending artery (arrows) in (**A**) 5-day-old newborn with double outlet right ventricle and in an (**B**) 8-month-old male infant with transposition of the great arteries and dextrocardia.

**Figure 3:**
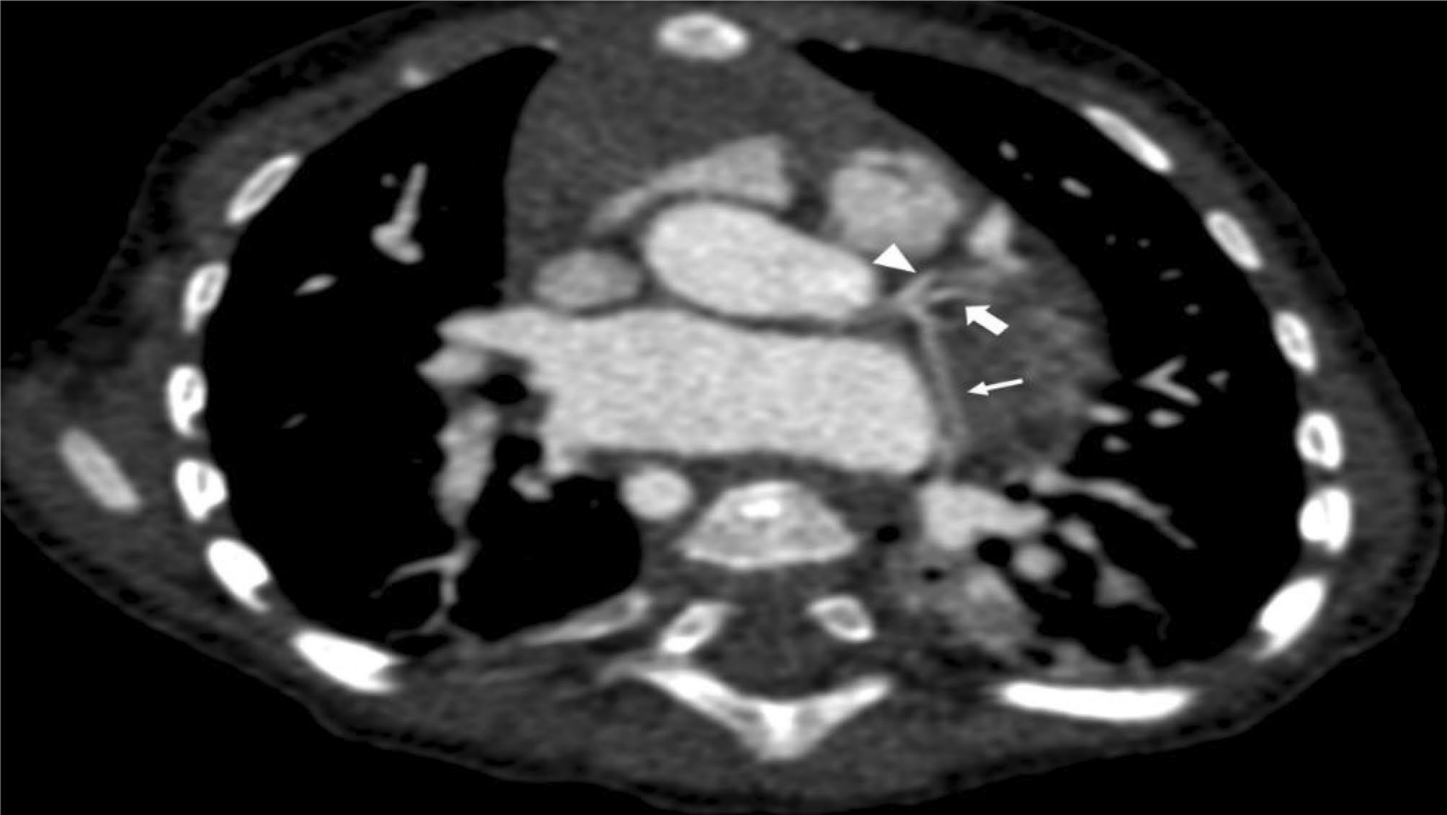
Double angulated reconstruction demonstrating a trifurcation of the left main coronary artery giving rise to the left anterior descending artery (arrowhead), a left median branch (thick arrow), and the left circumflex artery (thin arrow) in a 5-month-old female infant with a tetralogy of Fallot.

**Figure 4:**
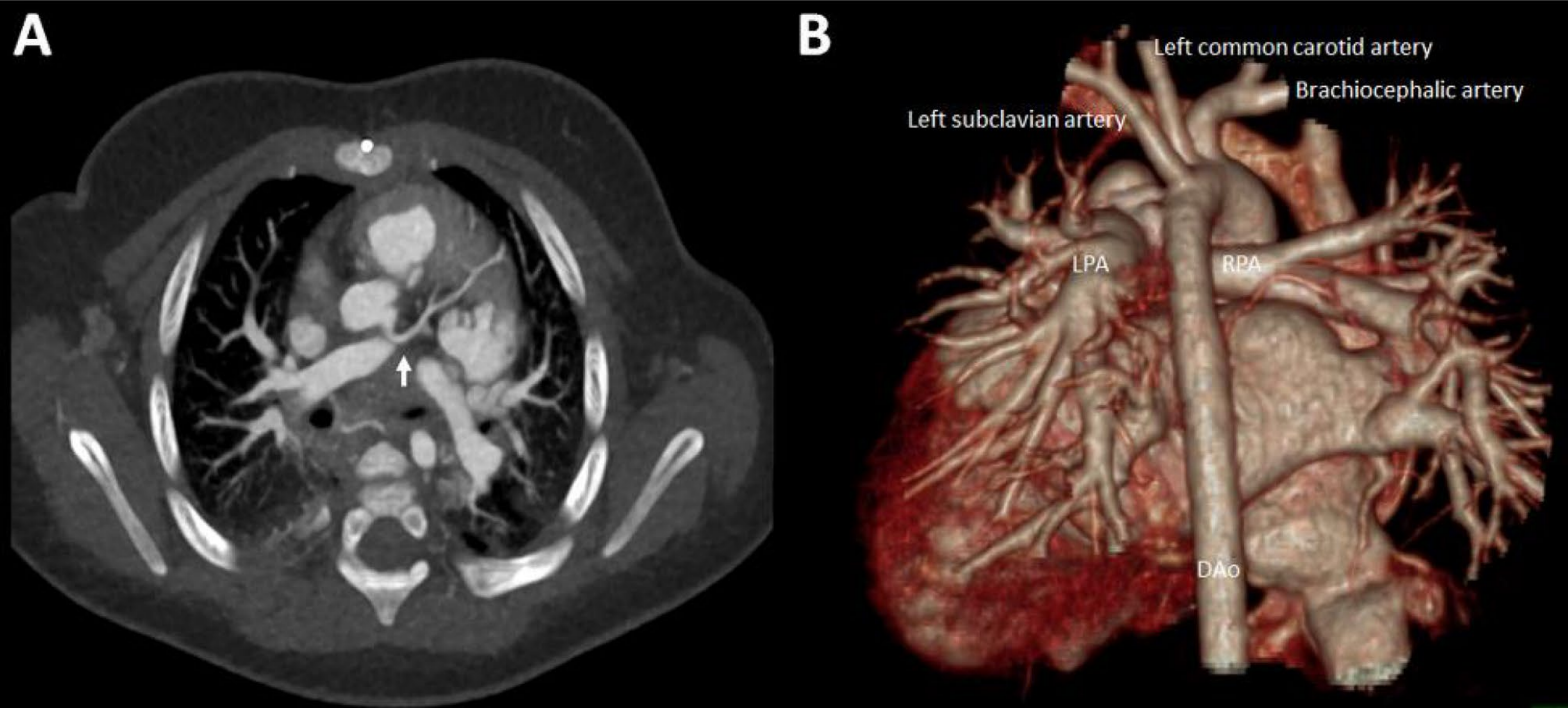
Pulmonary vessel anatomy in an 8-month-old female infant with a tetralogy of Fallot demonstrating good delineation of the left anterior descending coronary artery (**A**; arrow). (**B**) Dorsal view of a 3-D reconstruction of the heart and the great vessels and pulmonary veins. LPA: Left pulmonary artery. RPA: Right pulmonary artery.

**Figure 5:**
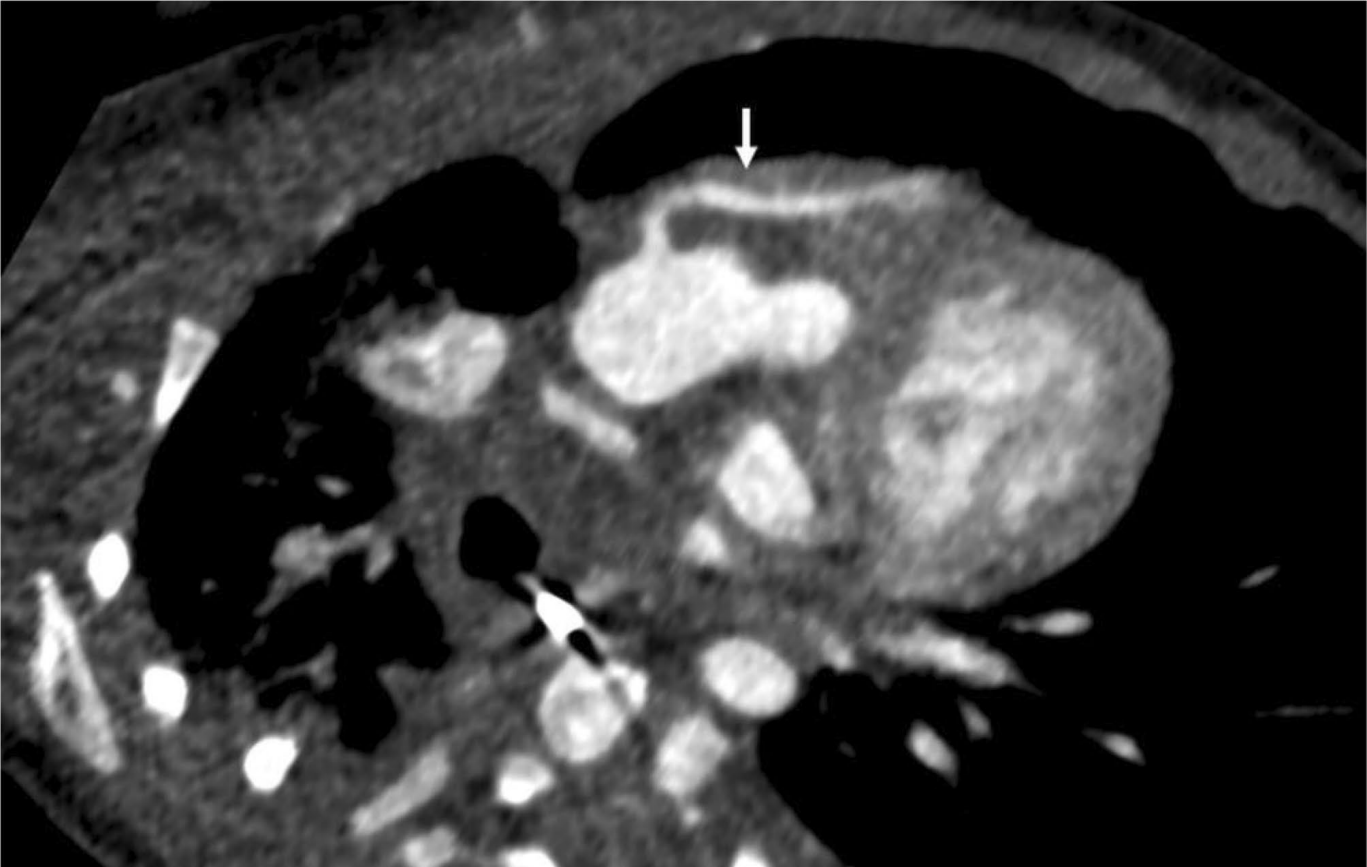
Detailed 3D angulated reconstruction visualizing the right main coronary artery (arrow) in a 2-month-old male newborn with Taussig-Bing syndrome.

Twenty-seven patients, six female (22%) and 21 male (78%), were retrospectively analyzed. CCT was performed for pre-operative assessment and for the evaluation of potential major aortopulmonary collateral arteries (MAPCAs) in hypoplastic left heart syndrome (HLHS; n = 6, 22%), Tetralogy of Fallot (FOT; n = 5, 19%), pulmonary atresia (n = 5, 19%), total anomalous pulmonary venous return (TAPVR; n = 4, 15%), double outlet right ventricle (DORV; n = 4, 15%), single coronary ostium (n = 1, 4%), transposition of the great arteries (TGA; n = 1, 4%), and aorto-left ventricular tunnel (ALVT; n = 1, 4%). The median age was 109 days (IQR 6–199). The median CTDI_vol_ was 0.34 mGy (IQR 0.22–0.51) while the median DLP was 5.2 mGy*cm (IQR 3.5–7.8). The calculated ED resulted in a median value of 0.21 mSv (IQR 0.14–0.30) after using a tissue specific conversion factor (k) of 0.039 for a pediatric thorax under the age of 0 years calculated from a 32 cm phantom. All performed CCT scans were able to fully answer all clinical questions (3 points on a maximum 3 point-scale with an interclass correlation coefficient of 1.00). Seventy-seven percent of the scans (n = 21) had an image quality score of excellent or good (15 to 12 points) while the lowest score was eight (poor quality, n = 1, 4%). Examples of acquired image quality are provided in Figs. 2, 3, 4, 5.

Subjective image quality score was correlated using Pearson’s correlation and found significant associations between CNR (r = 0.63, *p* =  < 0.001), heart rate (r = − 0.43, *p* = 0.02), and applied mAs (r = 0.43, p = 0.03). SNR did not show a correlation with image quality (r = 0.38, p = 0.05). The interclass correlation coefficient was excellent, with an agreement of 0.94. A detailed summary of the findings is summarized in Table 3.

**Table 3:** Patient characteristics and findings.

Parameters	Values
Heart rate, bpm	120 (35)
Scan length, mm	92 (27)
kV	70
mAs (total)	168 (74)
Scan initiation at	70% of RR Interval
CTDI_vol_, mGy, median (IQR)	0.34 (0.22–0.51)
DLP, mGy*cm, median (IQR)	5.2 (3.5–7.8)
Effective dose, mSv, median (IQR)	0.20 (0.14–0.30)
Descending aorta contrast enhancement, HU	548 (138)
SNR	16.2 (10.0)
CNR	18.2 (6.7)
Image quality score (max. 15, min. 3)	13.3 (2.1)
Clinical questions answered score (max. 3)	3 (0)

All values are expressed as mean with standard deviation in brackets unless otherwise noted. SNR and CNR was calculated at the descending aorta and intercostal muscles at the level of the pulmonal trunk. The image quality score was assessed on a 5-point scale for three categories: artefacts, noise, and vessel evaluation. The individual scoring was performed as follows: 1: not assessable, 2: poor quality (severe artefacts, excessive noise, questionable coronary artery delineation), 3: adequate quality (moderate artefacts, moderate noise, proximal coronary arteries are delineated), 4: good quality (minor artefacts, low noise, coronary arteries clearly delineated), 5: excellent quality (no artefacts, no noise, clear visualization of distal coronary artery segments). Subjective image quality score was based on a 0 to 3 scale: 0: non-diagnostic images, 1: some clinical questions could be answered, 2: most clinical questions could be answered, 3: all clinical question could be answered. BPM: Beats per minute. CTDI_vol_: Computed tomography dose index-volume. DLP: Dose length product. HU: Hounsfield units. SNR: Signal to noise ratio. CNR: Contrast to noise ratio.

## Discussion

This study was able to demonstrate that sufficient diagnostic imaging quality could be achieved in a pediatric CHD cohort employing high pitch single phase prospective gated imaging with comparably low radiation dose. CCT offers excellent spatial resolution and allows for superior assessment of extracardial structures when compared to echocardiography, however, at the cost of ionizing radiation. The use of CCT in the adult population has been extensively documented but the additional ethical and procedural constraints in the pediatric population have led to a sparsity of thorough studies for pediatric CCT imaging^12–14^. One of the main arguments for the reserved use of CCT in the pediatric population is the increased cancer risk associated with ionizing radiation for this population. Tachycardia with heart frequencies over 120 bpm, as typically found in infants, usually necessitating retrospective spiral acquisition to limit motion artefacts is another challenging requirement in pediatric cardiac imaging. Recent developments in CT technology (i.e. third generation dual source CTs, ultrasensitive detector technology, high pitch techniques) pave the way for a broader use of CCT in the pediatric population with high temporal resolutions and reduced radiation^15–17^. Conventional single source CTs suffer from information gaps if the pitch is increased beyond its associated detector size. DSCTs can utilize two detectors to cover these gaps and thus sufficiently increase pitch to values of up to 3.4^15,16^. Previous studies have highlighted the need to reduce radiation exposure in CHD patients as patient lifetime risk of cancer is markedly increased^18,19^. DSCT provides good spatial resolution, fast acquisition times, and the ability to reconstruct images in three dimensions.

In this current study, we were able to expand on the above mentioned technological advancements regarding radiation dose, achieving a relatively low ED for cardiothoracic CT while still being able to adequately answer all clinical questions. This feat was achieved by employing a high pitch prospective ECG triggered “FLASH” protocol on a DSCT which allows for the acquisition of the whole heart within one cardiac cycle. A median dose of 0.2 mSv is equivalent to approximately 37 days of background radiation (assuming a yearly background radiation of 2.4 mSv)^20^. The accurate representation of the coronary anatomy is vital in CHD patients, thus as in previous studies it was used as a quality reference standard ^9^. However, in most CHD cases, the complete depiction, including the distal sections of the coronary arteries is not needed as most pathologies are located in the proximal segment (e.g. anomalous ostia). Reliable high-quality depiction of the entire course of the coronary arteries would require high dose retrospective gating. The employed prospective gating protocol allowed for adequate depiction of the proximal course of all coronary arteries in all included patients.

Cardiac imaging for elevated heart rates has been shown to deliver superior image quality at end systolic phases (31–47%)^21^. Optimal gating can be difficult when imaging the entire thorax, especially when scans are commenced distant from cardiac structures. This is especially true at variable heart rates. In this study, total thoracic acquisition was performed. Image acquisition was commenced above the subclavian veins at 70% of the R–R interval. Even with high pitch (737 mm/s) and low acquisition times (average of 0.12 s) the progression of the cardiac cycle during the scan must be taken into consideration. Therefore, choosing a diastolic phase for scan initiation may be superior when the entire thorax is imaged. The employed contrast agent injection protocol enabled sufficient visualization of cardiac structures as well as thoracic vasculature without the need for contrast triggering, additionally reducing radiation dose due to the lack of bolus tracking. By utilizing diluted contrast agent and employing comparably slow injection velocities, allowing for delayed contrast infusion, simultaneous arterial and venous opacification was achieved. Higher concentration rates of contrast agent (e.g., 75% contrast agent and 25% saline solution) and faster infusion rates may be employed if coronary anatomy or only arterial depiction is explicitly required. Slower infusion rates render bolus tracking unnecessary in most cases, as scans can be commenced shortly after infusion has completed and both venous and arterial structures are already opacified. Consequently, we were able to achieve even lower effective doses than other current DSCT studies. A study by Yang et al. observed an ED of 2.85 ± 2.03 mSv in children 4–12 months old^22^. When compared to a paper by Wang et al. (average ED of 0.45 ± 0.23 mSv), we were able to achieve half the average ED with a smaller standard deviation (0.24 ± 0.12 mSv)^23^. Nie et al. had similar findings to us with a mean of 0.29 ± 0.08 mSv^24^. When compared to single source CTs, the estimated effective dose was nearly 75% lower (Le Roy et al.; 1.0 ± 0.3 mSv)^9^.

In accordance with other similar studies, we found a negative correlation between heart rate and the image quality score (r = − 0.43, *p* = 0.02), confirming that a lower heart rate leads to an improved image score^25,26^. CNR also, unsurprisingly, correlated with image quality (r = 0.63, *p* =  < 0.001).

Current papers describe an average reduction in heart rate of around 19 beats per minute or 23% under beta-blockers^27^. However, the mean age in the aforementioned study was 13 years old. The included patient cohort of the current study had a median age of 109 days with an average heart rate of 120 ± 35 beats per minute. Our experience was that for even small reductions in frequency, high doses of beta-blockers are required for such a young patient collective. Furthermore, pre-medication with beta-blockers prolongs sedation times and is not possible in all patients. Thus, we do not feel the use of beta-blocker is justified in this collective as the benefits of slightly decreased heart rates do not outweigh the risks of unwanted side effects and prolonged sedation times. Cardiac MRI and CCT have their respective use-cases. MRI is used primarily for function and myocardial assessment while CCT remains the modality of choice for vessel visualization as well as preoperative assessment for CHDs, for example in tetralogy of Fallot^28^. Unless newer MRI techniques can achieve the same excellent spatial resolution and fast acquisition times of CCT, CCT will remain a key diagnostic tool in cardiac imaging. This study is limited by its relatively low number of included patients (n = 27) and the diversity of the CHDs within the group (eight different defects were described).

## Conclusion

Dual source high pitch CT protocols combined with custom contrast agent injection protocols in pediatric patients with CHD delivered sufficiently high diagnostic imaging quality combined with low submilisievert radiation doses. Prospective high pitch imaging is a reliable method for depiction of cardiac anatomy even in very young pediatric CHD patients with elevated heart rates.

## Data Availability

Due to local privacy laws, our data security provider is unable to provide generated or analyzed datasets as theoretically there is a risk of identification of personal information in pseudo-anonymized CT datasets.
